# Exploring the Moderation and Mediation Effects in Addressing the Main Determinants of Income Inequalities in Supporting Quality of Life: Insights from CEE Countries

**DOI:** 10.3390/ijerph19148555

**Published:** 2022-07-13

**Authors:** Adriana AnaMaria Davidescu, Tamara Maria Nae, Margareta-Stela Florescu

**Affiliations:** 1 Department of Statistics and Econometrics, The Bucharest University of Economic Studies, 010552 Bucharest, Romania; 2 Department of Education, Training and Labour Market, National Scientific Research Institute for Labour and Social Protection, 010643 Bucharest, Romania; 3 Doctoral School of Economics, Bucharest University of Economic Studies, 010552 Bucharest, Romania; tamara.nae@economie.ase.ro; 4 Research and Analysis Department, Ministry of Finance, 050706 Bucharest, Romania; 5 Department of Administration and Public Management, Bucharest University of Economic Studies, 010374 Bucharest, Romania; margareta.florescu@ari.ase.ro

**Keywords:** inequality determinants, quality of life, well-being, mediation effect, moderation effect, CEE countries, panel data analysis

## Abstract

Income inequality has become an increasingly pressing economic and social problem in Europe, especially in emerging countries with more significant inequalities than the EU average. The high-level inequality persistence can decrease well-being by accentuating the shortcomings at the household level, increasing poverty and social exclusion, generating political instability, leading to a decline in social cohesion, and, finally, a weakening of the Union as a whole. In this context, the paper aims to identify the main determinants of income inequality across the CEE countries and their significant implications in supporting the quality of life and well-being, highlighting the mediation and moderation effects. The analysis focuses on emerging European countries, using panel-based data analysis for ten EU countries covering 2008–2019. The empirical findings highlighted the importance of the minimum wage, high-tech exports, the degree of economic openness, the quality of institutions, and education spending in reducing income inequality. On the other hand, the proportion of the population with a higher education level and the interaction between official and unofficial economies led to income inequality. Therefore, to increase the quality of life, it is mandatory to decrease inequalities. Thus, fewer people will be at risk of living a less qualitative life. The empirical results also proved that the informal economy and the share of people employed in industry exhibited mediating roles. In contrast, the economic growth, the urbanization degree, and the share of people employed in services exhibited moderating roles. Additionally, we also tested the impact of the income inequality determinants of the quality of life, the empirical results supporting the influence of minimum wage, employment with tertiary education, government effectiveness, the degree of economic openness, and education expenditures.

## 1. Introduction

Improving the quality of life and living conditions has been an important goal of European integration and cohesion processes, which are often identified in the fundamental and reform treaties of the European Union (Treaty of Rome, 1957; Maastricht Treaty, 1992) [1,2]. Over time, it has been shown that inequality is a phenomenon that reflects the level of well-being of a society, which is evidenced by its inclusion as a monitoring indicator in the dashboard attached to the European Pillar of Social Rights.

The importance of studying inequalities is provided on the one hand by the concerns expressed by international institutions about their persistence and growth, being considered the main problem of our times, and on the other hand by the effects of the pandemic shock, with the exacerbation of inequalities being discussed more and more often. The consequences of the growth and persistence of inequalities are summarized by Deaton (2017) [3], as follows: Inequality hampers the reduction of poverty, but also development; As income inequality increases, various associated social problems arise, such as declining life expectancy, declining welfare of the population, especially among children, which in turn leads to declining educational performance or even dropping out of school.

The central objective of this paper is to identify the determinants of income inequality, so that the results of this analysis can recommend a set of measures that will help policy makers in the process of reducing income inequality and thus helping to increase the quality of life and living conditions in the analyzed countries.

Over time, several factors were identified that can influence inequalities. However, it should be noted that they are not the result of a single phenomenon, but of an intertwining of phenomena driven by the interaction of social, geographical, economic, historical, and political aspects (Galasso, 2014) [4]. There are determining factors because of geography, gender, ethnicity, family background, etc. The inequalities generated by these factors are called by Galbrait (2016) [5] absolute inequalities; they cannot be easily eliminated and cannot be eliminated permanently and can only be mitigated under bold regulations and changing perceptions of differentiated issues.

Although there is a broad literature that explores the determinants of income inequality in general, very few of these studies explore the main implications of those determinants in supporting the quality of life in Central and Eastern European countries (CEE). In this context, the paper aims to identify the main determinants of income inequality across CEE countries and their significant implications in supporting the quality of life, highlighting the mediation and moderation effects. We empirically tested the impact of different groups of determinants, using panel data analysis, and explored the mediation and moderation analysis for CEE countries covering the period 2008–2019.

Our study contributes to the existing literature in several ways. Firstly, we offer a comprehensive perspective on the main determinants of income inequality specially designed for CEE countries, which has not previously received due attention. To our knowledge, the research contributes to a relatively limited number of studies dealing with this topic at the level of this group of countries. Secondly, special attention has been attributed in the study to the impacts of the informal economy mediating the relationship between economic growth and income inequalities, and their main implications in supporting the quality of life. Thirdly, appropriate policies and implications will be designed to sustain the main strategies in supporting the quality of life and mitigating income inequalities. Thus, filling the gap in income inequality literature for the group of CEE countries, for which the body of literature is still inconclusive, and justifying the need for further research.

The paper is organized as follows. The introduction initiates the reader in the topic of income inequality, pointing out the research’s relevance and highlighting the main contributions to the literature. Section 2 presents an overview of the most relevant determinants of income inequality in the literature, highlighting how the inequalities were affected by the pandemic shock and how the literature has reflected this. Section 3 presents the data and research methodology and sheds light on the discrepancy in income distribution among the CEE countries. Section 4 presents the main empirical results, incorporating the impact of COVID-19 on the income inequality among CEE countries, using a simulation forecasting scenario. The paper ends with the most relevant conclusions and recommendations for further research.

## 2. Exploring the Main Determinants of Income Inequalities and Their Implications for Supporting the Quality of Life

The research on the quality of life relates more to an overarching theoretical framework than it does to any one notion of welfare or well-being. Most of the numerous definitions and concepts of quality of life that are contained within this framework acknowledge the multidimensional nature of living situations as an important factor. The quality-of-life paradigm has provided the groundwork for a substantial amount of research on the cognitive and emotional aspects of pleasure and fulfillment, including contentment with life in general, as well as satisfaction with life domains. As a result, the quality of life is intimately tied to other (psychological) aspects of one’s subjective well-being, such as fears, dangers, and attitudes, as well as their relationships to other factors, the various phases, and the characteristics of one’s personality. The investigation of the quality of life is founded on techniques from a variety of fields of study, as well as normative applications for the upkeep and enhancement of living situations. As a result, individual-level research on inequality, poverty, and multiple deprivations is linked to quality-of-life approaches; social-level research examines the functioning of social and political institutions and the preservation of living conditions; and national-level and global-level research examines social and political indicators used widely to promote and establish fairer and better living conditions around the globe, Krause (2016) [6].

According to the UNDP (2011) [7], high and growing inequality can hinder and even stop progress towards the reduction in poverty, given the growth, inequality, and poverty links in a nation. Rising inequality is a fundamental source of domestic financial instability that is often associated with unfavorable growth, poverty, and distribution implications.

The research that was conducted by Kakwani and Son (2016) [8] demonstrates that inequality has a negative impact on a variety of aspects of well-being. According to the findings of this investigation, efforts should be made to reduce inequality to enhance the general well-being of a society. To make it possible for people to live better lives, public policies need to assist those who are at the very bottom of the distribution and enhance their access to economic possibilities, such as education, health care, and basic infrastructure.

Comparing the experiences of the United States and Britain concerning the progression of earnings and the impact of income inequality on death rates, Deaton and Paxson (2001) [9] discovered that there is no evidence to suggest a connection between income disparity and mortality rates. The primary purpose of this study is to investigate the relationship between economic disparity and well-being. This study will investigate whether inequality has a negative impact on wellbeing, and, if so, to what degree. The purpose of this study is to suggest that the price of income disparity is significantly higher than has been projected in the previous research. According to the findings, income disparity has a considerable negative impact on a variety of aspects of well-being.

According to Wilkinson and Pickett (2009) [10] (p. 1), “The benefits of greater equality tend to be largest among the poor but seem to extend to almost everyone,” the editorialists noted, adding that “a more equal society might improve most people’s quality of life”.

The post-socialist integration of Central and Eastern Europe into the EU is one of the success stories of European development. Since joining the EU, these countries have seen increasing economic convergence, changes in socio-economic indicators, and improvements in the natural environment. According to Łuczak and Kalinowsk (2020) [11], the population of the old EU countries are less severely affected by material deprivation than people living in the new member states. However, some of the challenges remain, such as economic and opportunity inequalities, political divergence, public governance issues, and declining population demographics.

The CEE countries are currently characterized by a level of inequality that is of concern for national institutions, but also for international ones. There is also an extensive body of literature confirming this (Sýkora, 1999 [12]; Golinowska, 2009 [13]; Mihaylova and Bratoeva-Manoleva, 2018 [14]; Wołoszyn and Wołoszyn, 2018 [15]; Szarfenberg, 2019 [16]; Černiauskas and Čiginas, 2020 [17]; Dudek and Szczesny, 2021 [18]; Wołoszyn et al., 2021 [19]).

Regarding labor market policies, in general, the minimum wage policy is of particular importance in Central and South-Eastern Europe, given that more employees are affected by this measure compared to the west or north of the continent (IMF, 2016) [20]. In addition, with large increases in the minimum wage in the region, the share of remunerated employees at the level of the minimum wage has risen, as they are particularly concentrated in the services’ sector (especially HoReCa) and light industry. In general, at the regional level, governments control the stability of the minimum wage, while specialized commissions of independent experts do not have a formal role, and objective mechanisms are often lacking (Concordia, 2020) [21]. The empirical evidence proved that the minimum wage fuels the shadow economy; aggressive increases in the minimum wage create a competitiveness problem in the context of a relatively high level of informal economic activities (Davidescu and Schneider, 2017) [22].

**Figure 1:**
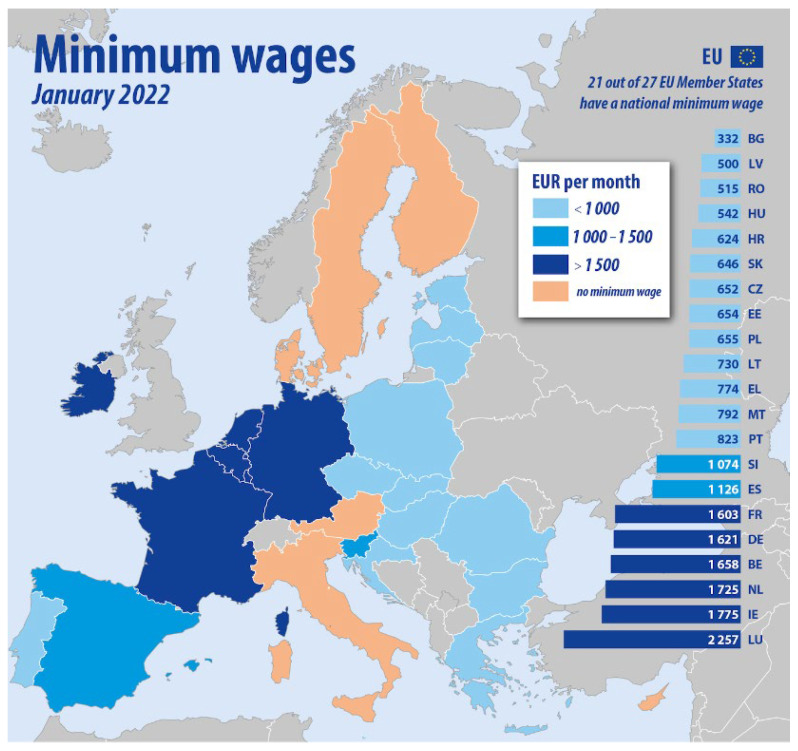
Minimum wage in EU. Source: European Commission, Eurostat [24].

A rise in the minimum wage (Figure 1) will, initially, create a loss of jobs, particularly among low-skilled employees. This is mostly due to the increased number of persons seeking employment at a higher income; as a result, the number of positions will fall, which directly impacts low-skilled workers (Maloney and Mendez 2004) [23].

According to a new survey from Eurostat, the statistical office of the European Union, regarding the map of the minimum wage, we can observe a segregation between East-Central Europe and Western Europe. However, there is also a difference inside the CEE countries, the highest minimum wage in emerging Europe is paid in Slovenia. As of January 2022, the minimum wage in the country was 1074 euros. At the other end of the spectrum, Bulgaria has the lowest minimum wage of all the emerging Europe’s EU members at 332 euros. Significant differences can be seen also for the eastern vs. western EU countries regarding the prevalence of the undeclared work, based on the data from Undeclared Work Eurobarometer (Table 1). The differences in participation in undeclared work across the EU regions revealed that the East-Central Europe countries exhibited a higher ratio of undeclared work (4.4% compared to 3.8%).

Analyzing the perceptions of those engaged and not engaged in undeclared work in both regions concerning expected sanctions, it was revealed that people involved in informal work from ECE countries perceived the sanctions and the risk as being lower than those not engaged in informal work or from Western Europe (30.7% of those participating in undeclared work from ECE countries consider that tax and social contributions will be due if caught, compared with only 23% of those participating in informal work from western countries). In addition, the tax morality of western countries’ individuals tended to be higher than that of the ECE countries’ individuals. Therefore, we can conclude that those individuals participating in undeclared work activities from East-Central Europe tend to have a lower level of expected sanctions and a smaller level of tax morale compared with those from western countries [22].

The literature has identified several factors that may influence income inequalities, most often the following: minimum wage (Litwin, 2015) [25]; human capital theory (Shapiro, 2006) [26]; issues related to globalization and development (Barro, 2000; Piketty, 2015; Mihaylova, 2016) [27,28,29]; technological progress (Kharlamova et al., 2018) [30]; the quality of institutions (Acemoglu and Robinson, 2013; Ostry et al., 2018) [31,32]; the share of the informal economy (Schneider, 2000; Dell‘Anno, 2008; Amarante and Arim 2017) [33,34,35]; as well as social spending (Jianu et al., 2021) [36].

Well-designed labor market policies and institutions can reduce inequality, so a relatively high minimum wage can play an equalizing role in distributing labor income and supporting the quality of life. However, its effect may be the opposite if the level is too high. This measure may reduce employment, which diminishes its effect on reducing inequalities (OECD, 2012) [37]. Many studies have shown that an increase in income inequality is associated with the inefficiency of labor market regulations, as well as a low minimum wage (Nolan, 2016; Bubbico and Freytag, 2018) [38,39]. The skills acquired through choices and education determine a certain income range. The rarer the skills, the higher the chance of getting a job that will result in an above-average salary level, and the more common the skills, the chances are reduced. Thus, the human capital theory emphasizes that young people increase their future earnings by giving up possible current earnings and continuing their studies. It has also been argued that education, especially higher education, is one of the main factors determining the differences in income distribution with a significant positive impact (Rodriguez-Pose and Tselios, 2009) [40]. At the same time, Mincer (1991) [41] identifies that an increase in the share of people with higher education leads to a decrease in salary for this category of employees, leading to an improvement in terms of income distribution. Some of the studies conclude that the link between education and income inequality is ambiguous (Földvari and Van Leeuwen, 2011) [42]. This makes it relevant to review and summarize the empirical evidence of the link between higher education, used as a proxy for human capital, and income inequality.

The development level of the economy is one of the most studied determinants in terms of the impact of the income distribution, the biggest leap since the controversial theory of Kuznets (1995) [43]. According to his hypothesis, inequality is low in pre-industrial societies, where most of the people live at subsistence levels. The starting point of this theory is the agrarian society, based on family farms and agricultural properties, which characterizes the rural part of a country. However, as industrialization began, cities are formed around factories. The gaps are starting to widen, as factory wages become more attractive than those for agricultural workers. Therefore, the cities will be richer than rural areas. The earnings continue to rise as more and more specialization emerges among the industrial workers. Kuznets considers that inequality will increase to a certain point where there is a general balance between the urban and rural populations. Once that point reaches a maximum value, as the urban population grows (the rural population goes/moves to the urban areas), the difference between the rural and urban becomes less important, and inequality will automatically reduce. The relationship between economic growth and income inequality is not linear, so research has yielded different results. One of the problems of inconclusive results is which econometric model was used.

Under the development umbrella, we find the degree of economic openness in the literature. According to Beaton et al. (2017) [44], those states with a higher level of economic openness tend to have a higher standard of living and a lower level of income inequality. While using the panel data method for the period 2000–2014 in CEE countries, Neagu et al. (2016) [45] identified a positive relationship between income inequality, the degree of economic openness, and foreign direct investment (FDI), with the education level showing an equalizing effect on income distribution. Mihaylova (2016) [29] found that the share of FDI has the potential to impact income distribution, but the effect differs depending on the education level and the economic development of the host countries. Another study, which includes FDI (Halmos, 2011) [46], tests the relationship between FDI, exports, GDP, and income inequality in CEE countries, identifying a positive and significant relationship between rising income inequality and rising FDI stock levels. The study results also show that higher high-tech exports (a proxy for technological progress) harms income inequality. Rodriguez (2020) [47] analyzed the relationship between globalization and income inequality, considering the differences in development level. The study focused on the effect of the degree of economic openness, FDI inflows; remittances received, and the rate of import tariffs on the Gini coefficient, using a panel data analysis. The author concluded that trade liberalization increases inequality in developed and developing countries. At the same time, FDI inflows are associated with greater inequality in developing countries, the effect being negative for developed and positive for developing countries.

According to Litwin (2015) [25], the declining share of the employed population in the industrial sector harms income inequalities, as people migrate to either the service sector or the agricultural sector, characterized by lower wages. This phenomenon leads to an increase in income inequality through two channels: (i) unskilled workers in the industrial sector are generally better paid compared to those in the service sector; (ii) this negative effect has been amplified by the decrease in unionization, given that the unions, through bargaining power, have significantly contributed to the increase of wages for unskilled workers.

Technological progress has been identified as a determinant of income inequality, but the impact varies. Bound and Johnson (1992) [48] tested the effect of technological change, among other variables, on income inequality. The main conclusion was that several factors contributed to the increase in inequality, but the technological revolution had the most prominent effect. Technological development requires a higher level of education for employees, thus creating a higher demand for skilled workers, which leads to an increase in income inequality. Milanovici (2016) [49] pointed out that the technological changes in recent decades have given an advantage to the category of workers familiar with computer work and information technology, while the low-skilled jobs have been relocated to developing countries. Iammarino et al. (2018) [50] noted that technological innovation has led to the concentration of technology-based sectors in large metropolitan areas, favoring the mobility of highly skilled employees, while in industry, low-skilled labor is underutilized. In addition, Kharlamova et al. (2018) [30] analyzed how technological change affects income inequality in European countries, resulting in the following: (i) the Central European countries and the United Kingdom reached a level of development and redistribution in the economy so that a change in labor productivity is not associated significantly with a deepening of income inequality; (ii) peripheral countries, due to their significant dependence on the larger economies and a lack of developed redistribution mechanisms, are significantly affected by technological changes; (iii) the more economically developed a country is, the lower the impact of technological changes on income inequality; (iv) the deeper the income inequality in a country, the more responsive it is to technological change, but the impact on inequality can be both positive and negative.

Most of the European countries face some degree of corruption and informal economies. A study conducted on a significant sample of countries (Dobson and Dobson, 2012) [51] identified a positive empirical link between the level of corruption, inequality, and the informal sector. The results support the intuitive argument about the role of the informal sector in explaining the trade-off between corruption and inequality. The data suggest that the marginal impact of corruption on inequality is reduced as the informal sector grows larger. Once the informal sector accounts for just over one-fifth of GDP, reducing corruption is no longer effective in reducing inequality. The authors explained how an increase in the level of corruption in countries with weak institutions and where the informal sector is significant can reduce inequality but draw attention to the fact that the level of corruption could increase uncontrollably. Countries may freeze in the future with a growing informal sector and an inefficient institutional framework. Moreover, the informal sector is, by its nature, unregulated, and many workers (adults and children) face dangerous exploitation and working conditions that worsen the general well-being of the population.

Jianu et al. (2021) [36] estimated, for both the developed and developing EU countries, the impact of certain determinants on income inequality, including education level, the unemployment rate, social spending, and income inequality in previous years (historical). The study’s main results suggested that the relationship between historical income inequality and its current size was stronger in developing countries than in developed countries. Social spending has a greater potential to reduce income inequality in developed countries. A positive relationship exists between higher education and income inequality in the developed EU countries, while a negative relationship has been identified in developing countries, explaining that access to higher education is higher in developing countries, providing opportunities to increase revenue. In developed countries, where skilled labor is better paid, increasing access to higher education can widen the income gap.

**Figure 2:**
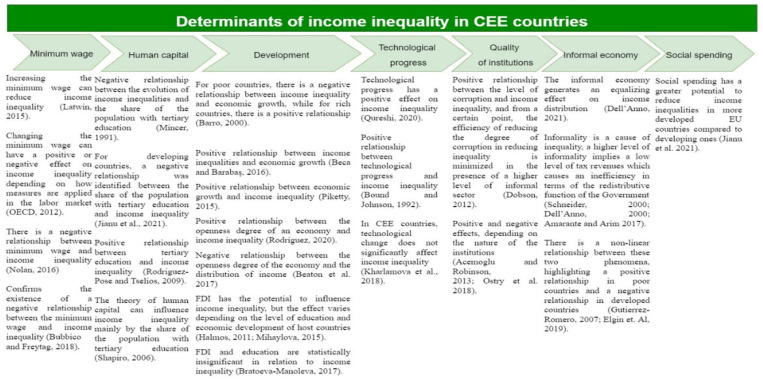
Determinants of income inequality in CEE countries [25,26,27,28,29,30,31,32,33,34,35,36,37,38,39,40,41,44,46,47,48,51,52,55,56,59,60].

The informal sector is an important part of the economy (the relative size of the informal sector accounts for more than 30% of aggregate economic activity in developing countries and almost 20% in developed countries). The labor market plays a major role in production and income generation and job creation for the low-skilled and disadvantaged. However, according to Dell’Anno (2021) [52], in addition to the beneficial effects on income and employment distribution, informality raises problems for public finances. It influences official statistics, reducing the effectiveness of redistributive policies. Despite the importance of the link between income inequality and informality, the literature is relatively small, identifying three types of links, as follows: (i) informality is determined by the level of income inequality, Chong and Gradstein (2007) [53] found that income inequality, in particular in conjunction with the quality of institutions, is a statistically significant and substantially robust determinant of the relative size of the informal sector, Mishra and Ray (2014) [54] also identified a positive impact; (ii) informality is a cause of inequality, due to the fact that a higher level of informality implies a lower level of tax revenue which causes an inefficiency in the redistributive function of the state (Schneider, 2000; Dell’Anno, 2008; Amarante and Arim 2017) [33,34,35]; (iii) there is a non-linear relationship between these two phenomena (Gutierrez-Romero, 2007) [55], highlighting a positive relationship in poor countries and a negative relationship in developed countries (Elgin et al., 2019) [56]. According to the literature, there is a positive relationship between income inequality and the informal economy. However, this link is caused by changes in GDP, rather than changes in the level of the informal economy (Dell’Anno, 2016) [57]. An increase in the minimum wage may be considered as a long-term support factor for the informal economy (Davidescu and Schneider, 2017) [58]. A synthesis of the specialized literature for CEE countries was designed, according to Figure 2, to understand better the main pillars and possible determinants of income inequality.

The structure of the expected impact for each variable, according to the theory, is presented in Appendix A, in which the mediation and moderation relations are also identified.

Based on the theoretical considerations, the following research hypotheses were highlighted:

**Hypothesis** **1** **(H1).** *The informal economy mediates the relationship between the formal economy and income disparities;*

**Hypothesis** **2** **(H2).** *The share of people employed in industry mediates the relationship between the degree of economic openness and the income inequality;*

**Hypothesis** **3** **(H3).** *Economic growth moderately affects the relationship between social expenditures and income inequalities;*

**Hypothesis** **4** **(H4).** *Urbanization moderately affects the relationship between minimum wage and income inequality;*

**Hypothesis** **5** **(H5).** *The employed population in the service sector moderately affects the relationship between the share of people employed in industry and income inequalities. More in-depth, it is worth mentioning that decreasing the share of people employed in industry combined with an increase in the demand for unskilled jobs in the service sector leads to a rise in income inequalities;*

**Hypothesis** **6** **(H6).** *The union density rate moderately affects the relationship between the share of people employed in industry and income inequalities. Assuming that trade unions were one of the essential tools for raising the wages of unskilled workers, a reduction in the unionization rate leads to an increase in inequalities.*

## 3. Data and Methodology

The importance of analyzing the determinants of income inequality lies mainly in identifying policies that prevent social challenges and improve income distribution, so that a larger share of the population is included in economic and social activities. In such a way, the standard of living of all of the citizens will improve, recovering and balancing the economic and social situation, increasing cohesion, and increasing the resilience of the economies to future asymmetric shocks.

Following the paper’s main objectives, the empirical analysis focused on the analysis and identification of the main determinants of income inequality among the ten CEE countries, excluding Croatia due to lack of data being the most recent country to join the European Union in 2013.

The empirical model is built using regression analysis on panel data, based on annual data covering 2008–2019.

In order to explore the potential determinants of income inequality, different specifications of the model have been tested, the general specification of the model being the following:

(1) $$\begin{array}{c} {\mathrm{Gini}}_{\mathrm{it}}=\beta_{0}+\beta_{1}{\mathrm{minimuwage}}_{\mathrm{it}}+\beta_{2}{\mathrm{humancapacityproxy}}_{\mathrm{it}}+\beta_{3}\\ {\mathrm{economicdevelopmentproxy}}_{\mathrm{it}}+\beta_{4}{\mathrm{technologicalprogressproxy}}_{\mathrm{it}}+\beta_{5}\\ {\mathrm{qualityofinstitutionsproxy}}_{\mathrm{it}} \end{array}$$

where: *i* = number of cross-sections and *t* = time used; *β*_0_—constant coefficient; *β*_(1–9)_—the coefficients of the determinants of income inequality.

Therefore, in the specifications of the models, we used seven classes of potential determinants of income inequality (minimum wage; human capital; economic development; technological progress; quality of institutions; informal economy; and social spending), including also additional control variables: (i) the share of persons employed in the service sector, because they tend to be better remunerated compared to those in the industry sector; (ii) the percentage of the population living in urban areas, to which are added the suburbs, because the minimum wage has different effects on employees, depending on the environment of origin (Wu et al., 2006) [61]; (iii) inflation, because, according to Monnin (2014) [62], low inflation rates are associated with higher income inequality; as inflation increases, inequality decreases, reaches a minimum with an inflation rate of about 13%, and then begins to grow again.

A detailed description of the variables is provided in Table A1 of Appendix B, the data sources being the database of the European Commission, Eurostat; the World Bank database and The Global Economy database that selects data from the following official sources World Bank, International Monetary Fund, the United Nations, and the World Economic Forum.

As the main limitations of the empirical research, we can mention: the lack of data availability for Croatia; the lack of recent estimates for the size of the shadow economy in CEE countries, the last estimate being provided by Medina and Schneider (2018) [63] at the level of 2015, as a consequence, within the research we used the last estimate for the period 2015–2019; due to the lack of data for the period 2008–2011 in the case of the innovation index, we used the value from 2011 for this period; data for the share of high-tech exports were available only until 2018, therefore, we also kept the same value for 2019; for the union density rate and the collective bargaining coverage rate, for the years with missing data, we assigned the values of the available last year.

In the first stage, we used the ordinary least squares method, cross-section and period fixed effects models, for our estimations. The temporal effects included in the models were aimed at capturing, over time, the income inequality that is common to all of the CEE countries. Testing of the Redundant Fixed Effects was used to decide which models are suitable for modeling our data set (fixed effects, periodic effects, cross-sectional effects, or both). The Hausman test was then used to identify whether a fixed-effect model (FEM) or a random effect model (REM) is more appropriate. A low probability in the Hausman test suggests using FEM (fixed effects models), while a high probability in the test emphasizes REM (random effects models).

The model type depends on the potential correlation of the explanatory variables with the unobservable effects (if the unobservable effects are uncorrelated with all of the explanatory variables, it is recommended to use models with REM effects). The empirical results of the Hausman test showed that the random effects estimator was consistent, with a probability very close to 1. Therefore, different specifications of the potential determinants of income inequality were estimated by assuming random effects, both spatial and temporal, using the Panel EGLS (two-way random effects) method.

We also tested the consistency of random effects by applying the Breusch–Pagan Lagrange (LM) multiplier that helped us to decide between regression of random effects and a simple OLS regression. The cross-sectional dependence was tested using three tests: Breuch–Pagan LM; Pesaran scaled LM; and Pesaran CD, while the homoscedasticity was tested using Panel Heteroskedasticity LR test, and the normality of residuals with the Jarque–Bera test. The empirical results for all of the models showed, at a significance level of 1%, that the random effects both in cross-section and period were suitable.

The problem of cross-sectional heteroskedasticity was addressed using standard corrected heteroskedasticity errors which improves the standard estimator errors, without changing the values of the coefficients. Durbin–Watson statistics were used to test for the presence of residual autocorrelation. All of the proposed econometric models were estimated using the E-Views 9.0 software package (IHS Global, Irvine, CA, USA) [64]. The goodness of fit of the models was evaluated using adjusted R2, RMSE and the model’s standard error. In contrast, the validity of the models was tested using the Fisher test.

Moderation occurs when the relationship between two variables (the dependent and independent variables) depends on a third variable, called a moderator. The effect of this moderator acts on the intensity or direction of the relationship between the dependent and the independent variables.

Using information from literature studies, we tested the mediator and moderator role of various variables, with a significant impact on income inequality.

**Figure 3:**
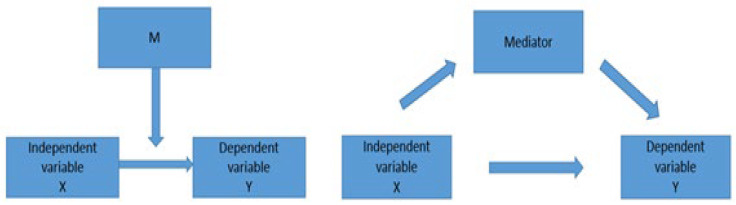
Mediation and moderation effects between dependent and independent variables. Source: Muller, Judd and Yzerbyt (2005) [65].

Mediation, on the other hand, is used to investigate the underlying mechanism or process by which one variable impacts another one (Figure 3).

In the research, the following mediation/moderation effects on income inequality were empirically tested:

- Mediation effects:
  ✓ The share of the informal economy mediates the direct relationship between the official economy and income inequalities;
  ✓ The share of people employed in industry mediates the relationship between the degree of economic openness and income inequality.
- Moderation effects:
  ✓ Economic growth moderately affects the relationship between social expenditures and income inequalities;
  ✓ Urbanization moderately affects the relationship between the minimum wage and income inequality;
  ✓ The employed population in the service sector moderately affects the relationship between the share of people employed in industry and income inequalities;
  ✓ The union density rate moderately affects the relationship between the share of people employed in industry and income inequalities.

Once the main determinants of income inequality were identified, it becomes essential to empirically identify which of those determinants manifested a significant impact on the quality of life. The most comprehensive index that captures the quality of life at European level is the index of social progress; it is based on the definition according to which social progress is the capacity of a society to meet the basic human needs of its citizens, establish the building blocks that allow citizens and communities to enhance and sustain the quality of their lives, and create the conditions for all individuals to reach their full potential (SPI Report, 2021) [66].

The Social Progress Index (SPI) was developed based on the theories of Amartya Sen, Douglass North, and Joseph Stiglitz and measures the quality of life and social well-being of citizens in 163 countries, based on the analysis of three main dimensions. The methodology consists of assigning a score for articles in the basic needs category (basic nutrition and health care, water and sanitation, shelter, and personal safety), for articles in the welfare category (access to basic knowledge, access to information and communications, health and welfare, quality of the environment), and for the category of opportunities (personal rights, freedom and personal choice, inclusion, access to advanced education) (The Economist, 2013) [67].

Thus, four additional models were estimated, one for each of the three pillars and the synthetic measure (SPI) to identify which of the income inequality determinants support the quality of life across the CEE countries.

## 4. Empirical Results

The importance of analyzing the determinants of income inequality lies mainly in identifying policies that prevent social challenges and improve income distribution, so that a larger share of the population is included in economic and social activities, improving the standard of living of all citizens, recovering, and balancing the economic and social situation, increasing cohesion, as well as increasing the resilience of the economies to future asymmetric shocks.

Different model specifications were examined, with the statistical impact of various determinants tested, and the variables with no statistically significant impact eliminated. The random-effects estimator was consistent, according to the empirical results of the Hausman test. The results of the LM test, which were highly significant at 1% and addressed significant disparities across the countries, also verified the use of random effects. The empirical findings are presented in Table A2 from Appendix B, with the minimum wage, the share of high technology exports, degree of economic openness, institutional quality, corruption perception index, education spending, the ratio of the population employed in higher education, economic growth, and the size of the informal economy all preserving statistical significance in all of the specifications.

The negative impact of the minimum wage on income inequality is a notable finding, which is corroborated by studies by Litwin (2015) [25], Nolan (2016) [38], Bubbico and Freytag (2018) [39], especially for the emerging economies. Increases in the minimum wage would help minimize income inequality by increasing income for low-paid workers. However, there is still no consensus on the minimum wage’s effects on income inequality, with some authors claiming that it has a favorable effect (Ferreira et al., 2014) [68]. The idea of re-distributivity, which involves the transfer of capital from other sections of the economy to low-income employees, and the theory of marginal productivity are the theories on which the relationship between the minimum wage and income inequality is founded. Due to the low level of access to higher education and the low-income features of the studied countries, the ratio of the employed population with higher education had a positive and statistically significant impact on income inequality. The findings are according to Rodriguez-Pose and Tselios’ research (2009) [40]. The advancement of technology has had a detrimental and severe impact on income inequality. Despite contrary or ambiguous findings in the literature, high-tech exports may be a significant component in decreasing inequality in the CEE economies.

Although the literature stipulated both forms of income distribution consequences, in our situation, the economic development proxies (GDP per capita or economic growth) had a favorable impact, which was also supported in the literature by Piketty (2015) [28] and Kuznets studies (1995) [43]. According to Beaton et al. (2017) [44] study, the degree of economic openness resulted in a decline in inequality for emerging market economies and an increase (however not statistically significant) in inequality for advanced economies. Only when market inequality is taken into account does the negative effect of economic openness on inequality for emerging markets become significant, implying responsibility for redistributive policy.

The collective bargaining rate harmed income inequality, with collective bargaining affecting the wages and benefits of those not directly covered by an agreement when employers meet collective bargaining standards, or at least improve their compensation and labor practices beyond what they would have provided in the absence of collective bargaining. In contrast, the union density rate had no significant impact, implying a potential relationship between the two. In addition, the moderation effect on the relationship between the share of people employed in industry and income inequalities has been statistically invalidated, leading to the invalidation of Hypothesis H6.

Foreign direct investments, contrary to the findings of Halmos (2011) [46], Mihaylova (2016) [29], and Bratoeva-Manoleva (2017) [59], have no substantial impact on income inequalities.

Another important finding is that the quality of institutions has a significant impact on income inequality, as evidenced by Acemoglu and Robinson (2015) [31]. They found that an improvement in institutional regulations combined with increased corruption control decreased income inequality. At the same time, an increase in corruption perception leads to a reduction in income inequality, considering that an increase in corruption perception in countries with weak institutions and a large informal economy, such as CEE countries, will result in a reduction in income inequality through increased employment (Dobson and Dobson, 2012) [51].

The empirical study revealed an important finding: the informal economy has a positive and statistically significant impact on income inequality, mediating the relationship between economic development and income distribution. It is generally recognized in this set of countries that the official economy impacts the informal economy and that the informal economy impacts income inequality, maintaining this function of mediation. The findings are partially validated by the research of Gutierrez-Romero (2009) [55] and Elgin et al. (2019) [56], which found that the informal economy has a favorable influence in emerging nations and a detrimental impact in rich countries, with the former being also confirmed by Dell’Anno’s study (2021) [52].

As the number of employees in manufacturing decreases, so does the degree of unionization. This phenomenon increases income inequalities in two ways: (i) unskilled workers in the manufacturing sector are generally better paid than those in the service sector; (ii) this negative effect has been amplified by the decline in unionization, given that unions have significantly contributed to the increase in unskilled workers’ wages through bargaining power.

**Figure 4:**
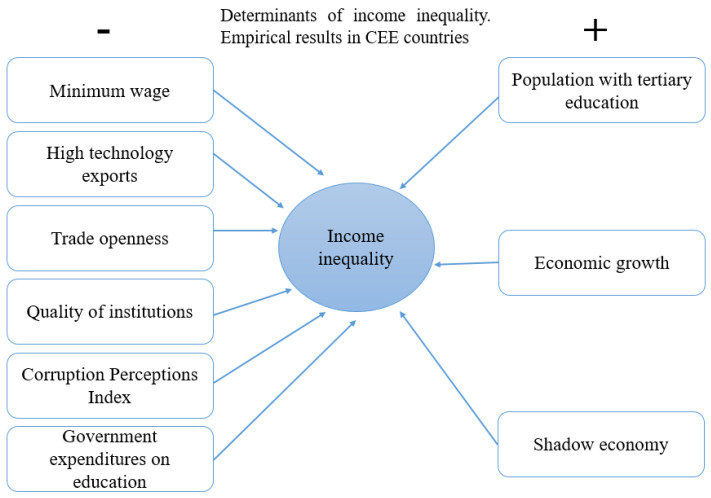
Determinants of income inequality. Empirical results in CEE countries.

The employed population in the service sector exhibited a moderation effect on the relationship between the share of people employed in industry and income inequalities, validating the Hypothesis H5. Therefore, we proved that decreasing the share of people employed in industry, combined with an increase in the demand for unskilled jobs in the service sector, leads to a rise in income inequalities. All of the statistically significant determinants of income inequality are displayed in Figure 4.

All of the estimated models were statistically valid, with a high level of goodness of fit. The models were corrected using standard heteroskedasticity errors without modifying the coefficient values, based on the improvement of standard estimator errors. The examination of residuals revealed that the residuals are not normally distributed, emphasizing the connection between the cross-sections and the presence of heteroskedasticity.

Thus, the minimum wage, the share of high-tech exports, the openness of the economy, government efficiency, corruption control, and the share of education spending decrease the income inequality, whereas the share of people with higher education and the share of the informal economy is leading to growing inequalities in CEE countries. Thus, raising the minimum wage, increasing economic openness, increasing high-tech exports, intensifying corruption control, and improving institutional quality would reduce income inequality. In contrast, increasing the share of people with higher education in developing countries would increase inequality by providing better-paid jobs (significant differences between unskilled and low-skilled people compared to high-skilled people).

The empirical results indicated that income inequality in CEE countries is influenced by the following core determinants that kept their statistical significance in all of the specifications: minimum wage; the share of high technology exports; degree of economic openness; quality of institutions; perception index of corruption; spending on education; the share of the population employed in higher education; economic growth; and the share of the informal economy. These variables were grouped according to the impact manifested in two groups, as shown in Figure 4.

Furthermore, using these previously identified determinants, we tested their impact on the quality of life, keeping in the models only those ones that were statistically significant. The empirical results are presented in Table A3 from the Appendix.

## 5. Discussion

One of the first remarkable results, which is in line with previous studies identified in the literature for emerging countries (Litwin, 2015; Nolan, 2016; Bubbico and Freytag, 2018) [25,38,39] concerns the negative impact of the minimum wage on income inequality; the sign of the variable was preserved in all 16 of the estimated models. The results are also statistically significant, most at a significance level of 1%.

The share of the population with a higher education shows a strong positive impact on income inequality, which was unanimously preserved throughout the testing process, also having a statistical significance of 1%. According to the theory, an explanation of this relationship may be given by the low level of accessibility to higher education due to the low incomes characteristic of the countries analyzed. The same positive impact was identified by Rodriguez-Pose and Tselios (2009) [40].

Another variable, a component of technological progress, was shown to have a negative, significant impact on income inequality. The share of high-tech exports may be a key element in reducing inequalities in CEE countries, despite the opposite, or inconclusive results, identified in the literature.

As expected, economic development generates both a positive and a negative impact on income distribution, depending on the proxy included in the analysis. In the case of CEE countries, for the period 2008–2009, GDP and the economic growth rate show a positive impact, a result also identified by (Piketty, 2015; Beca and Barabaș, 2016) [28,60] and the degree of economic openness shows a negative impact, which is also supported in the analysis of Beaton et al. (2017) [44]. The literature explains that the depth of income inequality in a country determines the intensity of the response to technological developments. The impact on income inequality can be both positive and negative. At the same time, despite the significant results identified in the literature (Mihaylova, 2016; Halmos, 2011; Bratoeva-Manoleva, 2017) [29,46,59], the share of foreign direct investment did not have a significant impact on income inequality in the countries analyzed, for the period 2008–2019.

According to the literature, another notable result is the significant negative impact of the quality of institutions on inequalities (Acemoglu and Robinson, 2015) [31]. This result suggests that improved institutional regulation and increased control over corruption could reduce revenue inequality in the analyzed countries. At the same time, an increase in the level of corruption leads to a decrease in income inequalities. The theory can explain that an increase in corruption in countries with weak institutions in a high informality context can reduce inequality by increasing employment, at least in CEE countries (Dobson and Dobson, 2012) [51].

**Figure 5:**
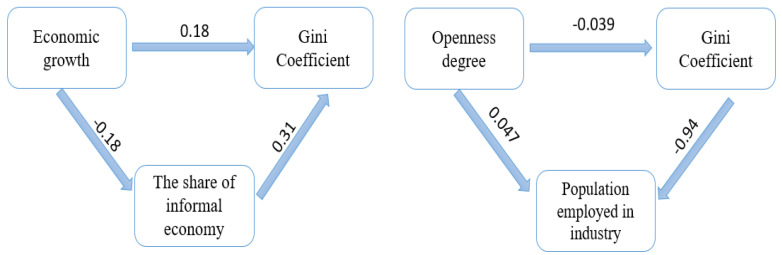
Mediation effect in exploring the determinants of income inequality.

The shadow economy acts as a mediator in explaining the dynamics of the relationship between the official economy and income inequality, validating Hypothesis H1. On the other hand, the share of manufacturing employment act as a mediator in explaining the relationship between the degree of economic openness and income inequality, validating the research Hypothesis H2 (Figure 5). Thus, increasing the degree of economic openness leads to a decrease in the share of the population employed in manufacturing. Therefore, increasing imports will reduce demand for the manufacturing sector, increasing income inequality.

In contrast with Dell’Anno’s (2021) [52] results, which proved the negative impact, the empirical results pointed out a positive influence of informality on income inequality. The results are partially validated by the studies of (Gutierrez-Romero, 2009; Elgin et al., 2019) [55,56] which stipulate that the informal economy positively impacts emerging countries and negatively impacts in rich countries. However, the share of the informal economy plays a mediating role in the positive relationship between economic growth and income distribution, confirming Hypothesis H1 (Figure 5).

Therefore, the degree of economic openness shows a negative impact on the Gini coefficient (the coefficient having a value of −0.39). However, following the moderation effect given by the share of the employed population in industry, the negative impact is significantly higher. This relationship is explained by the decrease in demand in the industrial sector due to increased imports.

The mediation effect is easy to observe in the two schemes, so that in the related economic growth–income inequalities, the share of the informal economy plays a mediating role (the coefficient of the direct relation, 0.18 being lower compared to that of the indirect relation, moderated by the third variable, 0.31). Similarly, the degree of economic openness has a direct negative impact of 0.039, while the negative impact of the indirect relationship, mediated by the share of people employed in industry, is 0.94. These results were obtained at the aggregate level for the CEE group of countries.

The potential moderation effects have been captured through hypotheses H3–H6 and are presented in Table A2 from the Appendix. The empirical results highlighted the validation of most of them, only except for H6, which was invalidated.

A significant result was identified on social spending, especially the share of education spending, which shows a negative result, in line with the recent analysis by Jianu et al. (2021) [36]. The empirical results proved the validity of research Hypothesis H3, confirming the moderation effect of economic development on the relationship between social expenditures and income inequalities.

The indirect impact of the degree of urbanization was a positive one, and statistically significant, having a moderating role on the relationship between the minimum wage and the level of inequality, leading to the validation of research Hypothesis H4. The results are supported by the studies of Wu et al. (2006) [61] and Litwin (2015) [25].

In addition, decreasing the share of people employed in industry, combined with an increase in the demand for unskilled jobs in the service sector, leads to a rise in the income inequalities; the employed population in the service sector exhibiting a moderation effect on the relationship between the share of people employed in industry and income inequalities, and validating the Hypothesis H5. The results are supported by the study by Litwin (2015) [25].

The decline in the number of jobs available in manufacturing has also resulted in lower unionization rates. A drop in unionization should increase inequality, since unions are one of the primary methods used to boost employees’ incomes with low levels of education and training. According to Freeman (1993) [69], decreasing unionization has been linked to greater inequality, even though the consequences were minor. We assume that there will be a general rise in income disparity because of a decreased supply of manufacturing employment, which would lead to an increase in demand for low-skilled service occupations and decreased unionization rates. However, the lack of statistical significance of interaction term between union density rate and share of the population employed in industry invalidated the potential moderation effect of the union density rate, invalidating the H6 Hypothesis.

Additionally, in order to support the link between income inequality and the quality of life, we tested the impact of the income inequality determinants highlighted by the optimal model (M14) on the quality of life, as it is captured by the Social Progress Index and its major pillars. Therefore, we tested the impact of the minimum wage, the degree of economic openness, government effectiveness, control of corruption, informality, economic growth, and education expenditure on all four proxies of life quality. We eliminated the variables without any statistically significant impact on quality of life from the models.

The empirical results pointed out very clearly that an increase in the minimum wage determines the increase in the overall level of social progress in total, but compared to the main three components of the SPI, we noticed that the impact is statistically significant only for the pillar of basic needs and fundamental well-being. A potential explanation for the lack of statistical significance of the pillar opportunities could be that they are not anymore connected to minimum wage, but with a level of respect for human rights and freedoms, tolerance in society and access to advanced education, aspects that are beyond the scope of the minimum wage level.

In addition, the increase in government efficiency has the potential to increase social progress, both overall and in its components, government efficiency considerations being closely linked to the reduction in income inequalities, through the efficiency of fiscal policy.

The degree of economic openness has a positive impact on both the SPI and the pillar of fundamental welfare, which suggests that an increase in the level of exports leads to economic growth, therefore a material welfare that can be reflected in an increase in living standards and, implicitly, on the quality of life.

The growth of the shadow economy causes a decrease in the SPI as it can have the maximum effect in ensuring the basic needs for a certain category of citizens (those without income or with limited income), but also undermines social progress by affecting the respect for fundamental rights and freedoms, both components of the opportunities pillar.

However, on the one hand, the shadow economy acts as a buffer for those marginalized groups of individuals, helping them to acquire additional earnings.

On the other hand, the shadow economy moderates the relationship between economic growth and fundamental well-being, the impact being negative in the presence of the shadow economy, or in other words, the shadow economy undermines the influence of economic growth in achieving the fundamental well-being.

La Porta and Schleifer (2014) [70], Gerxhani (2004) [71], and Schneider and Enste [33] all concluded that a large dimension of the unofficial economy could have negative externalities on the official economy. These negative externalities include absorbing capital assets and the labor force from the formal economy, limiting state resources, creating imbalances in the official statistics, and engaging in inequitable competition with businesses that operate in the formal sector and comply with tax payment legal regulations.

As a direct result of this, not only will the amount of money that is collected in taxes fall, but so will the standard of essential public goods and service. The transition of inputs (labor and capital) from the official economy to the informal sector during times of recession exemplifies the negative link that exists between the two economies. Because the informal sector uses the public infrastructure that is maintained by the contributions of formal companies and individuals, without having any participation in expanding these public goods, the informal sector creates a negative externality that has an impact on the efficiency of the formal economy (Loayza, 2016 [72]; Loayza et al. [73]).

Increased spending on education has a positive impact on the SPI’s pillar of opportunities, suggesting that greater state involvement in the education sector can drive social progress by providing opportunities for citizens.

Thus, amongst the determinants of income inequality, such as the minimum wage, employment with tertiary education, government effectiveness, the degree of economic openness, education expenditures bring their contribution to the improvement of the quality of life, in counterbalance to phenomena such as the shadow economy and its relationships with the main macroeconomic aggregates, which need further investigation and monitoring in order to achieve the desideratum of a better quality of life.

## 6. Conclusions

The current pandemic setting enhances the population’s susceptibility to being exposed to the hazards caused by the amplification of disparities. This phenomenon expresses itself most often among the nations with weaker labor market institutions. An urgent need is to examine the development of income disparity factors from 2008–2019. These factors are potent weapons that might help bring about more equitable income distribution.

This paper’s central objective has been to identify the main determinants of income inequality, exploring the mediation and moderation effects. The regression analysis based on panel data, based in turn on annual data covering the period 2008–2019, was applied to a sample of ten CEE countries, so that the results of this analysis can recommend a set of measures that will help policymakers reduce income inequality and thus help increase the quality of life and living conditions in the analyzed countries.

The research tested the significance of the seven potential pillars of income inequality, pointing out the influence of the minimum wage, the share of high-tech exports, the degree of economic openness, government effectiveness, control of corruption, and education spending in reducing income inequality.

While the proportion of people with higher education, together with the interaction between both economies (official and unofficial), led to an increase in income inequality, it is mandatory to decrease inequalities to increase the quality of life. Thus, fewer people will be at risk of living a less qualitative life.

An increase in the minimum wage improves the income of low-paid employees, hence lowering income inequality. The theories of re-distributivity and marginal productivity are the foundation of the link between minimum wage and income disparity.

An increase in economic openness led to a decrease in the proportion of the employed population in manufacturing. This phenomenon was caused by an increase in imports, which reduced the demand for goods produced by manufacturers, leading to an increase in income inequality.

The reduction in income inequality may be attributed to a strengthening of institutional rules and the tightening of controls over corrupt practices. Thus, an increase in the perception of corruption in countries characterized by weak institutions and a prevalent informal sector, as is the case of CEE countries, will cause a reduction in income inequality. Therefore, we can say that an increase in corruption leads to a decrease in income inequality (Dobson and Dobson, 2012) [51].

Additionally, education spending led to a decrease in income inequality, since higher education tends to reduce inequality in developing countries. A country’s income distribution is more unequal when access to and opportunities for higher education are more unequally distributed (Jianu et al., 2021) [36].

To fully understand the nature of the link between informality and inequality, it is necessary to include a third key component: the official economy. It is common knowledge that both economies are deeply intertwined, and that this intimate connection may foster either beneficial or harmful sorts of connections. Because it offers job options to those with modest levels of expertise, the informal sector increases human capital accumulation. This is particularly true in developing nations; informality-reducing policies based solely on tax reduction and enhanced enforcement of tax and regulatory rules may lead to disappointing economic outcomes for transitional and emerging countries. These outcomes include increased inequality and higher long-term unemployment rates (Dell’Anno, 2021) [52].

There was equal compensation among individuals with comparable abilities when there was minor informality in the workplace. The pay gap between the workers with high and low levels of competence shrank. As a result, the increased formalization of low-skilled labor contributed to a narrowing of the inequality gap.

The empirical exploration of the mediation effects on income inequality revealed that the informal economy was mediating the relationship between the official economy and income disparities, and the role of the people employed in industry mediated the relationship between the degree of economic openness and the income inequality.

Therefore, we can mention that people with low or no incomes participate in the informal economy, ensuring the increase in their disposable income and reducing the income gap. At the same time, as wages for jobs in industry are cheaper in developing countries, the growth of international trade has caused many of these jobs to move abroad, causing some displacement from production sectors in developed countries. Increased imports reduce demand in industry. So, trade does not directly affect income inequality but instead affects the size of the manufacturing sector, which then affects inequality.

In terms of moderation effects, the empirical results highlighted the role of economic growth in influencing the relationship between social expenditures and income inequalities, revealing that the higher the economic growth rate, the higher the social expenditures and the more they bear the burden of the population.

In addition, urbanization exhibited a moderation effect on the relationship between minimum wage and income inequality. In urban areas, increases in the minimum wage create effects of unemployment, especially for workers in low-income families. Hence, the relationship between the minimum wage and income inequality depends on the level of urbanization.

The employed population in the service sector exhibited a moderation effect on the relationship between the share of people employed in industry and income inequalities. Increased demand for unskilled (service) jobs are combined with lower wages for unskilled workers, thereby increasing inequality.

Considering all of these, the governments and institutions of Europe must give serious thought to the following proposals:

- Measures are taken to address the factors that contribute to income disparity. Following the results that were generated through the empirical analysis, the focus needs to be in both directions: on the one hand, improving the determinants leading to decreases in income inequalities, and on the other hand, monitoring those determinants leading to increases in income inequalities;
- Ex ante analyses to streamline and ensure transparency in legislative decisions. It is of the utmost importance that, prior to any legislative change that could influence public finances (for example, increasing the minimum wage), there be debates and consultations, but most importantly, an analysis of the impact that is anticipated;
- Increasing the quality of institutions. The efficiency of public programs in countries with weak institutions tends to be lower, contributing to greater inequality and poverty levels.

Therefore, the determinants of income inequality, such as the minimum wage, employment with tertiary education, government effectiveness, degree of economic openness, and education expenditures, all bring their contribution to the improvement of the quality of life, in counterbalance with phenomena such as the shadow economy and its relationships with the main macroeconomic aggregates, which needs further investigation and monitoring in order to achieve the desideratum of a better quality of life. Our results fill the gap in the literature, offering some innovative results in supporting the relationship between income inequality and the quality of life and can provide valuable information for the policymakers in addressing income inequality, which is the main objective of the 2030 Agenda.

The importance of quality-of-life analysis is increasingly addressed in many economic, social, and political changes. In further research, we aim to analyze this phenomenon from a specific perspective of the European Social Model. How it influences EU cohesion policy (represented by structural and investment funds) and the quality of life (represented by indicators of economic development and social progress) will remain an open question. Future research is needed to provide an answer, and the research will definitively target regional data for the CEE countries group.

## Figures and Tables

**Table 1:** Expected sanctions, detection risk and tax morale in East-Central Europe and Western Europe.

	East-Central Europe	Western Europe
Participation in undeclared work (%)	4.4%	3.8%
Sanctions (%)		
Tax or social security contributions due	30.7%	23%
Tax or social security contributions fine or prison	69.3%	77%
Tax morale index (mean)	4.23	3.65
No participation in undeclared work (%)	95.6%	96.2%
Sanctions (%)		
Tax or social security contributions due	30.4%	16.8%
Tax or social security contributions fine or prison	69.6%	83.2%
Tax morale index (mean)	2.67	2.11

**Figure A1:**
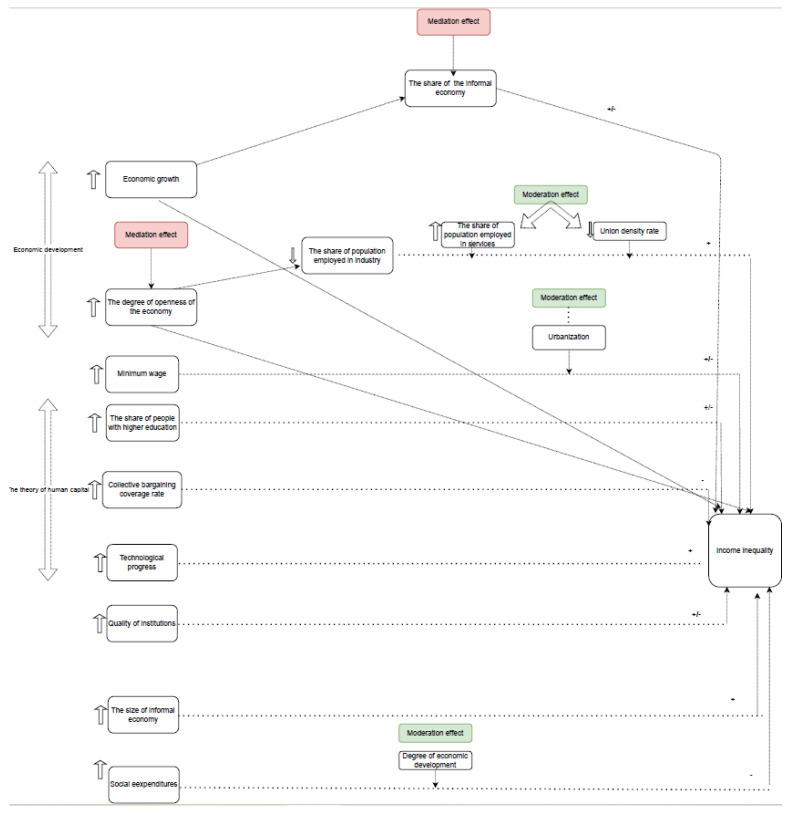
The main determinants of income inequality.

## Data Availability

Data can be available upon request.

## Appendix B

**Table A1:** Variables’ description and data sources.

Indicator	Definition	Source	Expected Impact on Inequality
Dependent Variables			
Gini Coefficient (GINI COEF.)	Gini coefficient calculated on disposable income (percentage values from 0 to 100, where 0 represents full equality and 100 represents full inequality)	Eurostat (ilc_di12)	-
Independent variables			
Minimum wage (MMWBI/MMWICS)	Minimum monthly salary, annual value is the average of annual data (purchasing power parity)	Eurostat (earn_minw)	-
	Minimum monthly wage as a share of average monthly earnings in industry, construction and services. (%)	Eurostat (earn_mw_avgr2)	-
Human capital theory			
Employed population with tertiary education (EM_ED_TER)	Share of the population employed with tertiary education in population aged 15–64. (%)	Eurostat (edat_lfse_03)	−/+
Union density rate ^1^ (%) (UNION_DENSITY)	Union density or union membership rate is the number of union members who are employed as a percentage of the total number of employees in a given industry or country.	ILOSTAT, ILO	
Collective bargaining coverage rate (%) (COL._BARGAIN)	The coverage rate of collective bargaining represents the number of employees whose salaries and/or conditions of employment are determined by one or more collective agreements as a percentage of the total number of employees. The coverage of collective bargaining includes, as far as possible, workers covered by collective agreements by virtue of their extension. Collective bargaining coverage rates are adjusted for the possibility that some workers may not have the right to bargain collectively on wages (for example, public service workers whose wages are set by state regulations or other methods involving consultations), unless in which it is otherwise stated in the notes.	ILOSTAT, ILO	
Economic development			
GDP per capita (GDP PER CAPITA)	The gross domestic product reported at no. of inhabitants, fixed base index 2010.	Eurostat (nama_10_pc)	(+/−)
Economic growth/cap. (EC. GROWTH PER CAPITA)	GDP growth rate, percentage change compared to the previous period, compared to no. residents.	Eurostat (TEC00115)	(+/−)
Openness of the economy (OPENESS)	The sum of exports and imports as a share of GDP. (%)	Eurostat (nama_10_gdp)	(+/−)
Share of foreign direct investment in GDP (FDI)	Foreign investment inflows directly reported by GDP.	Global Economy	(+/−)
The share of employees in industry (MANUF.)	The share of the population employed in industry.	Eurostat (lfsa_egan2)	-
Technological progress			
Share of high-tech exports (HIGHTECHXP)	Exports of high-tech products as a share of total exports. (%)	Eurostat (htec_si_exp4)	+/−
Quality of institutions			
Regualtory quality (REG_QUAL)	It reflects perceptions of the government’s ability to formulate and implement sound policies and regulations that enable and promote private sector development.	World Bank	(−)
Rule of law (RULE_OF_LAW)		World Bank	(−)
Government Effectiveness (GOV_EFFECT)		World Bank	(−)
Perceptions of corruption			
Corruption perception index (CPI)	Indicator of public sector corruption. The values of the indicator are determined on the basis of information from surveys and evaluations.	Global Economy	(−/+)
Control of Corruption (CONTROL OF CORR)	It reflects perceptions of the extent to which public power is exercised for private gain, including forms of corruption, as well as the “capture” of the state by elites and private interests	World Bank	(−/+)
Informal economy (%)			
Informal economy (%) (SHADOW_EC)	Informal economy as a share of GDP. (%)	Global Economy	(−/+)
Social expenditures			
Education expenditure (%) (ED_SPEND)	Share of education expenditure in GDP.	Eurostat (gov_10a_exp)	(−)
Health expenditure (HEALTH_SPEND)	Share of health expenditure in GDP	Eurostat (gov_10a_exp)	(−)
Control variables			
Urbanization degree (URBANISATION)	The share of people living in urban areas.	Eurostat (ilc_lvho01)	(+/−)
Employees in the service sector (%) (EMP_SEV)	Share of the employed population in the service sector (sum of the shares of economic activities in the field of services according to NACE Rev. 2)	Eurostat (lfsa_egan2)	
Harmonized index of consumer prices (HIPC)	Harmonized index of consumer prices, fixed base, 2010 = 100	Eurostat (prc_hicp_midx)	

^1^ A trade union is defined as a workers’ organization set up for the purpose of promoting and defending the interests of workers. This union density rate transmits the number of union members who are employed as a percentage of the total number of employees. For the purposes of this indicator, in particular, union membership excludes members of unions who do not have a paid job (self-employed, unemployed, pensioners, etc.), unless otherwise provided in the notes.

**Table A2:** Empirical results of income inequality determinants.

Variables	M1	M2	M3	M4	M5	M6	M7
Minimum wage	−0.02 ***	−0.004 ***	−0.005 ***	−0.004 ***	−0.007 *	−0.009 ***	−0.01 ***
Minimum wage (% median wage)							
Min_wage * urban	0.0001 ***						
Min_wage * Informality					0.0005		
Tertiary ed.(%)	0.47 ***					0.45 ***	0.28 ***
Coll. Bargaining (%)					−0.03 *		
Union density(%)							
Innovation index						−0.05	
High tech. exp. (%)							−0.079 ***
GDP per capita							0.08 ***
Economic growth	0.15 ***	0.14 ***	0.94 ***	0.17			
Economic openness	−0.07 ***	−0.06 ***	−0.06 ***	−0.05 ***	−0.01	−0.07 ***	−0.07 ***
FDI (%)							
Emp. in manufacturing (%)			−1.60 ***				
Emp in manufacturing * Emp in services		0.01	0.01 *	0.005	−0.01 ***		
Emp in manuf. * union density		−0.0001	0.0004	0.002	−0.002		
Regul. quality							
Rule of law	−3.58 ***	−0.23 *	0.02 *	−2.58 ***		−1.83 ***	
Gov. effectiveness					−1.69		−4.12 ***
Corruption Perception Index(CPI)							
Control of corruption							
Informal ec. (%)	0.05 **				−0.39 ***	0.064	
Informal ec. * Ec. growth						0.005 ***	
Educ. Expend.(%)		−1.39 **	−1.19 *			−0.84 **	−0.4
Health Expend(%)				0.26			
Educ. expend* Ec. growth			−0.13 ***	0.005			
Urbanization							
Emp. in services				−1.09 ***			0.20 ***
HIPC							−0.03
Constant	34.57	64.27	62.51	56.51	58.43	37.94	20.60
Obs.no.	120	120	120	120	120	120	120
F-test	127.99 ***	67.75 ***	66.78 ***	52.56 ***	7.47 ***	26.66 ***	102.81 ***
RMSE	2.08	2.08	1.98	2.19	1.38	1.85	1.64
S.E. of Reg.	2.15	2.16	2.07	2.29	1.44	1.92	1.71
R^2^	0.88	0.83	0.84	0.81	0.35	0.65	0.89
Adj.R^2^	0.88	0.81	0.83	0.79	0.30	0.63	0.88
Jarque–Bera	3.05 (0.21)	4.2 (0.12)	2.94 (0.22)	4.47 (0.10)	95 (0.008)	5.02 (0.08)	4.74 (0.09)
LM test for random effects Breusch–Pagan Two Sided	19.70 (0.00)	110.75 (0.00)	98.01 (0.00)	137.48 (0.00)	183.04 (0.00)	17.95 (0.00)	2.42 (0.10)
Breusch–Pagan LM	114.70 (0.00)	82.69 (0.00)	61.83 (0.04)	105.29 (0.00)	96.87 (0.00)	108.11 (0.00)	109.39 (0.00)
Pesaran Scaled LM	7.34 (0.00)	3.97 (0.00)	1.77 (0.07)	6.35 (0.00)	5.46 (0.00)	6.65 (0.00)	6.78 (0.00)
Pesaran CD	0.96 (0.33)	2.97 (0.00)	1.76 (0.07)	5.04 (0.00)	0.54 (0.58)	0.21 (0.83)	3.40 (0.00)
Panel Cross Section Heteroskedasticity LR test	55.09 (0.00)	61.28 (0.00)	58.77 (0.00)	81.30 (0.00)	87.62 (0.00)	58.50 (0.00)	35.11 (0.00)
**Variables**	**M8**	**M9**	**M10**	**M11**	**M12**	**M13**	**M14**
Minimum wage	−0.01 ***	−0.01 ***	−0.01 ***	−0.001 ***	−0.006 **	−0.001 ***	**−0.02 *****
Minimum wage (% median wage)							
Min_wage *urban		0.0001 ***	0.0001 ***	0.0001 ***	7.65 *	0.0001 ***	**0.0001 *****
Min_wage * Informality							
Tertiary ed.(%)	0.49 ***	0.29 ***	0.29 ***	0.30 ***	0.17 ***	0.32 ***	**0.50 *****
Coll. Bargaining (%)							
Union density (%)							
Innovation index	−0.08						
High tech. exp.		−0.004 ***	−0.003 ***	−0.004 ***	−0.006 ***	−0.005. ***	**−0.006 *****
GDP per capita							
Ev. growth							
Ec. openness	−0.07 ***	−0.05 ***		−0.04 ***	−0.04 ***	−0.05 ***	**−0.068 *****
FDI (%)	−0.01						
Emp. in manufacturing (%)		−0.51 ***	−0.53 ***	−0.48 ***	−0.63 ***	−0.48 ***	
Emp. in manufacturing * Emp. in services							
Emp. in manufacturing * union density							
Regul. quality	−2.35 **		−1.79 **				
Rule of Law		−1.06 **					
Gov. effectiveness				−2.44 ***			**−2.76 *****
Corruption Perception Index (CPI)						−0.07**	
Control of corruption					−2.92 **		**−0.07 ****
Informality (%)							
Informality * Ec. growth		0.003 *	0.003 **	0.003 *	0.003 *	0.003 *	**0.004 *****
Educ. Expend. (%)	−1.12 ***	−1.22 ***	−1.23 ***	−0.98 ***	−0.58 *	−1.22 ***	**−0.50 ****
Health expend. (%)							
Educ. Expend. * Ec. growth							
Urbanization							
Emp. in services							
HIPC							
Constant	43.12	50.98	52.46	48.04	49.53	52.51	**38.75**
Obs.no.	120	120	120	120	120	120	**120**
F-test	33.12 ***	94.65 ***	74.06 ***	78.56 ***	18.44 ***	94.21 ***	**77.51 *****
RMSE	1.87	1.70 1.77	1.63 1.70	1.61 1.69	1.37 1.43	1.67 1.75	**1.86** **1.94**
S.E. of Reg.	1.94						
R^2^	0.67	0.88	0.85	0.86	0.60	0.88	**0.86**
adj.R^2^	0.65	0.87	0.84	0.85	0.56	0.87	**0.85**
Jarque–Bera	4.79 (0.09)	2.16 (0.33)	3.44 (0.178)	2.65 (0.26)	11.02 (0.004)	2.24 (0.32)	**0.48** **(0.78)**
LM RE Breusch–Pagan Two Sided	17.71 (0.00)	8.71 (0.00)	3.64 (0.05)	3.15. (0.07)	6.84 (0.00)	6.86 (0.00)	**8.73** (**0.00)**
Breusch–Pagan LM	94.85 (0.00)	99.30 (0.00)	89.30 (0.00)	96.71 (0.00)	76.94 (0.00)	98.67 (0.00)	**119.23** **(0.00)**
Pesaran Scaled LM	5.25 (0.00)	5.72 (0.00)	4.67 (0.00)	5.45 (0.00)	3.36 (0.00)	5.65 (0.00)	**7.82** **(0.00)**
Pesaran CD	0.81 (0.41)	0.70 (0.47)	1.20 (0.22)	1.82 (0.06)	2.38 (0.00)	1.70 (0.08)	**1.46** **(0.14)**
Panel Cross Section Heteroskedasticity LR test	46.74 (0.00)	45.17 (0.00)	44.41 (0.00)	46.81 (0.00)	48.04 (0.00)	45.60 (0.00)	**54.92** **(0.00)**

Note: ***, **, * mean statistically significant at 1%, 5% and 10%; () represents the probability. Optimal model is marked in bold.

**Table A3:** The empirical results of the income-inequality determinants on the quality of life.

Dependent Variable	Basic Needs	Fundamental Well-Being	Opportunities	SPI
	Coefficient	Coefficient	Coefficient	Coefficient
Intercept	90.34512 ***	66.13353 ***	70.50241 ***	77.55094 ***
Minimum wage	0.004662 ***	0.0074 ***	−0.001238 *	0.003928 ***
Government Effectiveness	3.712574 ***		4.783452 ***	2.503778 ***
Employed population with tertiary education		0.192782 ***		
Trade		0.028504 ***		0.00916 ***
Shadow economy * Economic Growth		−0.001776 **		
Shadow economy				−0.04362 *
Education Expenditures			0.524479 *	
Hausman test prob.	0.55	0.65	0.18	0.71
R-squared	0.77	0.44	0.36	0.58
F-statistic	105.34 ***	24.08 ***	17.61 ***	43.27 ***
Prob(F-statistic)	0.00	0.00	0.00	0.00

Note: ***, **, * mean statistically significant at 1%, 5% and 10%; () represents the probability.
